# Association of the Ephreceptor Tyrosinekinase-Type A2 (*EPHA2*) Gene Polymorphism rs3754334 with Age-Related Cataract Risk: A Meta-Analysis

**DOI:** 10.1371/journal.pone.0071003

**Published:** 2013-08-16

**Authors:** Jin Yang, Jianfeng Luo, Peng Zhou, Qi Fan, Yi Luo, Yi Lu

**Affiliations:** 1 Department of Ophthalmology, Eye and ENT Hospital, Fudan University, Shanghai, China; 2 Department of Health Statistics and Social Medicine, School of Public Health, Fudan University, Shanghai, China; Sanjay Gandhi Medical Institute, India

## Abstract

**Background:**

Recent clinical studies have assessed the association of various polymorphisms on the ephreceptor tyrosinekinase-type A2 (*EPHA2*) with the risk for age-related cataract in populations of different ethnic/racial backgrounds, but inconsistent results have been obtained.

**Objective:**

This meta-analysis aimed to identify if any polymorphism(s) might be commonly present in different ethnic/racial populations in association with the age-related cataract risk.

**Methods:**

The PubMed and Web of Science databases (up to December 1, 2012) were searched for clinical studies on the association of *EPHA2* polymorphisms with the risk for age-related cataract. The polymorphisms that were assessed in all eligible studies were analyzed for their association with the risk for age-related cataract using different models.

**Results:**

Three studies were identified, which were conducted, respectively, on white Americans in the Unites States and on Asians in Indian and China. The polymorphism, rs3754334, was the only one studied in all these three studies and was therefore the focus of this meta-analysis. No publication bias or heterogeneity was found. Our analysis results demonstrated that rs3754334 was associated with the risk of any cataracts in the recessive (OR = 1.202, 95% CI: 1.051–1.375, P = 0.007) and Codominant (OR = 1.194, 95% CI: 1.035–1.378, P = 0.015) models, but its association with cortical or nuclear phenotype of age-related cataract was not evident.

**Conclusion:**

Polymorphism, rs3754334, might be a variant on the *EPHA2* gene that is commonly associated with the risk for age-related cataract in different ethnical and geographical populations.

## Introduction

Age-related cataract, also known as senile cataract, is a condition where cloudy deposits gradually accumulate on the crystalline lens of the eye in people aged 50 years and over. Although cataracts can be removed surgically, this treatment option may not be feasibly available to a large proportion of patients in resource-limited countries or regions with inadequate surgical services and high operation costs [1]. Moreover, patient outcome following cataract surgery varies considerably with the type of the surgery performed, the performer's surgical experience, and the presence of ocular comorbidities, and environmental and social conditions [2]. It is therefore not surprising that cataract remains the leading cause of vision impairment and blindness worldwide [3]. According to the latest information posted on the official website of Hellen Keller International (http://www.hki.org/working-worldwide/asia-pacific/china/) and a recent editorial article published in New England Journal of Medicine [4], cataract is responsible for at least 50% of the total (approximately 2.5 million) cases of blindness in China. As China's population is rapidly aging, age-related cataract is becoming an increasingly significant public health problem in the country.

The development of age-related cataract is a complex process, involving multiple factors. In the past few decades, extensive research efforts have been devoted to identifying risk factors and characterizing their nature and specific roles in the pathogenesis of age-related cataract worldwide. It is anticipated that identification and characterization of the major risk factors for age-related cataract may help doctors adopt individualized preventive and treatment measures. Although a spectrum of demographic, environmental, life style-associated, disease-related and miscellaneous factors have been proposed as risk factors for cataract, no associations between cataract and these putative risk factors except for increasing age have been consistently demonstrated [2]. In 2000, Hammond et al. reported the results of their analyses of 506 pairs of female twins in the New England Journal of Medicine that genetics, age and environment accounted for 48%, 38% and 14% of the variation , respectively, in the severity of age related nuclear cataract [5]. It is now increasingly accepted that genetics is the single most important factor in the development of cataract (accounting for at least 50% of the risk), followed by aging, while environmental factors are less important than thought [5], [6]. Accordingly, identification and characterization of variations in the genes key to the development of cataract have become a focus of numerous recent studies.

Eph-receptor tyrosine kinase-type A2 (*EPHA2*) is a member of the subclass A of the Eph subfamily of receptor tyrosine kinases [7]. Located on the short arm of chromosome 1 at position 36, the human *EPHA2* gene encodes a 976 amino acid, type-1 transmembrane protein with an extracellular NH2-terminal domain and a cytoplasmic COOH-terminal domain [8], [9]. Although the Ephs and their ligands, ephrins, were initially identified in the central nervous system [10], it is now evident that they are also present in a variety of other tissues including the ocular lens [11], [12] where they play important roles in the cellular communication [13]. Recently, the association of the various single nucleotide polymorphisms (SNPs) on the *EPHA2* gene with the risk for various subtypes of cataract has been assessed. However, the results from studies on different racial/ethnical populations in different geographic regions worldwide are inconsistent [14]–[16]. The present meta-analysis aimed to establish the true association between *EPHA2* SNPs and the risk for age-related cataract commonly present in different racial/ethnical population groups.

## Materials and Methods

### Literature search

The electronic databases of PubMed and Web of Science were last searched on December 1, 2012 using the following search terms: “cataract”, “*EPHA2*” or “Eph-receptor tyrosinekinase-type A2” and “genetic variant” or “polymorphism”. Articles that fulfilled the following four inclusion criteria were selected: (a) evaluation of the association between polymorphisms in the *EPHA2* gene and age-related cataract risk, (b) retrospective case-control studies or prospective cohort studies, (c) availability of data sufficient to estimate an odds ratio (OR) with a 95% confidence interval (95% CI), (d) publication in the English language with full text. There were no restrictions on sample size in the search. Clinical studies cited both in the selected articles and in relevant review papers were also retrieved. In cases of overlapping, only earliest studies were used. In cases of duplicated publication, only studies with the largest sample size were used.

### Determination of analysis variables

The following variables were extracted from each of the retrieved articles: first author's family name, publication year, country, numbers of cases and controls, ethnicity, phenotypes and genotypes. Phenotypes were categorized as cortical cataract, nuclear cataract, and any cataracts.

This extraction was performed by two of the authors (P. Zhou and J. Yang) independently. Disagreements were resolved by discussions between the two authors. If no consensus could be reached, a third investigator (Y. Lu) made the final decision.

### Statistical analysis

The primary measure of this meta-analysis was the strength of the association of age-related cataract with the SNP(s) of interest on the *EPHA2* gene. Four crude ORs and their 95% CIs for the association between the SNP(s) being evaluated and age-related cataract were calculated: (1) heterozygous versus common homozygous carriers, (2) rare homozygous versus common homozygous carriers, (3) rare allele carriers versus common homozygous carriers (dominant model), and (4) rare homozygous versus common allele carriers (recessive model). Heterogeneity among the included studies was assessed by the Q-test [17]. Dependent on the presence or absence of heterogeneity, a fixed-effects model (the Mantel-Haenszel method) or a random-effects model (the DerSimonian and Laird method) was employed. The possible publication bias was examined visually in a funnel plot of log [OR] against its standard error (SE), and the degree of asymmetry was assessed by Egger's test. Potential outliers (i.e., data points that are far outside the norm) were identified by a sensitivity analysis.

The statistical analysis software Stata/SE version 10.0 (Stata Corporation, College Station, TX, USA) was used. In all tests, differences were considered significant when P<0.05.

## Results

**Figure 1:**
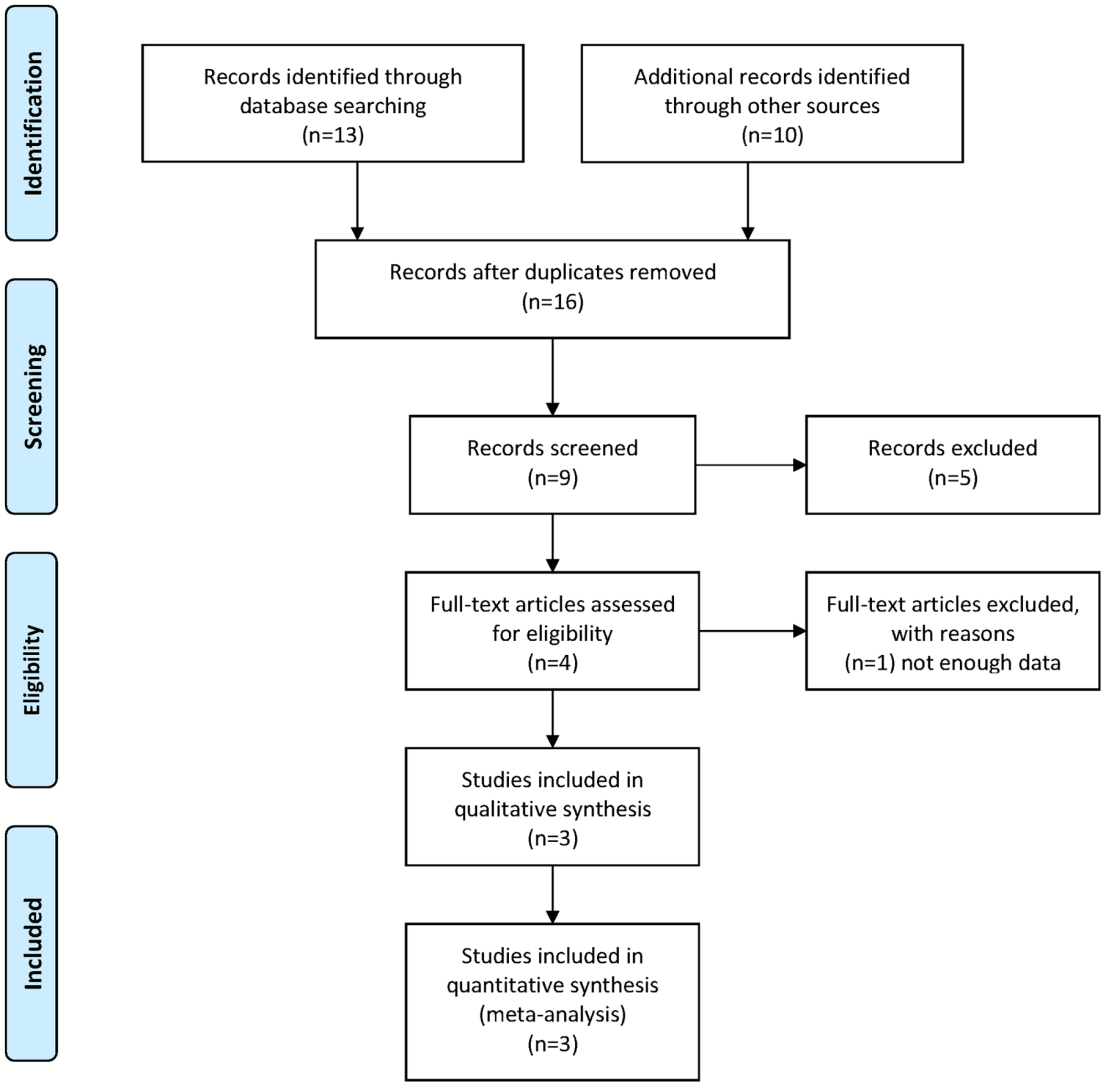
PRISMA flow diagram.

Three studies met the inclusion criteria and were included in this meta-analysis (refer to the Flow Diagram in Fig. 1). They were conducted, respectively, in the United States on white Americans of European origin [14] and in India and China on Asian populations [15], [16]. Comprising 1071 cortical cataract cases, 2600 nuclear cataract cases, 4776 any cataract cases, and 3616 controls, these three studies assessed more than eight SNPs in the *EPHA2* gene for their associations with age-related cataract. Among these SNPs, rs3754334 was the only one that was studied in all the three included studies and had adequate information for a meta-analysis. As a result, we focused on rs3754334 in this study. Summarized in Table 1 are the distributions of types (i.e., CC, CT and TT) of the rs3754334 variation in different phenotypes (i.e., cortical, nuclear and any cataracts) assessed in the three studies included in this meta-analysis.

**Table 1:** Summary of the three individual studies included in this meta-analysis with regard to the polymorphism, rs3754334, on the *EPHA2* gene in patients with age-related cataract and control subjects.

Phenotype	Year	First Author	Genotypes			MAF
			CC	CT	TT	
**Cortical**	2008	Shiels	47	43	17	0.36
	2011	Tan	301	111	10	0.16
	2012	Sundaresan	213	262	67	0.37
**Nuclear**	2008	Shiels	55	43	15	0.32
	2012	Sundaresan	1049	1106	332	0.36
**Any Cataract**	2008	Shiels	87	75	27	0.34
	2011	Tan	301	111	10	0.16
	2012	Sundaresan	1736	1846	583	0.36
**Control**	2008	Shiels	45	42	5	0.28
	2011	Tan	222	84	11	0.17
	2012	Sundaresan	1362	1460	385	0.35

MAF: minor allele frequency.

**Figure 2:**
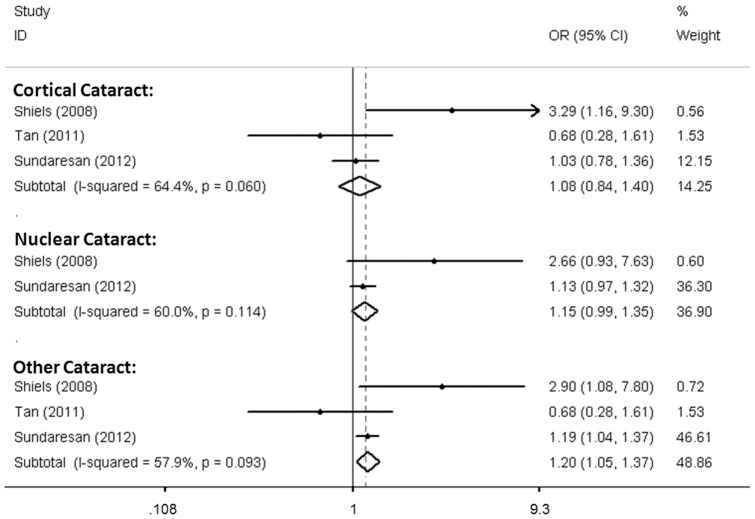
A forest plot, showing the relative strength of the association between rs3754334 with different types of cataract in the three studies in the recessive model.

Presented in Table 2 are results of the meta-analysis of the data pooled from the three included studies. No significant heterogeneity was detected for all variables assessed among the three included studies. The polymorphism rs3754334 was demonstrated to be associated with the “altered any phenotype” category of age-related cataract risk in both the recessive model (OR = 1.202; 95% CI: 1.051–1.375, p = 0.007) and the codominant model (OR = 1.194; 95% CI: 1.035–1.378, p = 0.015), but not in other genetic models. When analyses were performed on the phenotype subgroups, no association was evident between rs3754334 and cortical or nuclear age-related cataracts. Shown in Figure 2 is the forest plot, illustrating the relative strength of the association of rs3754334 with different types of cataract in the three studies in the recessive model.

**Table 2:** ORs and CIs for the polymorphism rs3754334 calculated based on different genetic models.

Analysis model	Pooled OR (95%CI)		
	Cortical	Nuclear	Any Cataract
Het vs. Common Hom	1.091(0.928–1.283)	0.978(0.876–1.092)	0.988(0.900–1.085)
*P/Ph*	0.292/0.662	0.691/0.594	0.805/0.962
			
Rare Hom vs. Common Hom	1.148(0.878–1.502)	1.143(0.969–1.348)	**1.194(1.035–1.378)**
*P/Ph*	0.313/0.078	0.113/0.161	**0.015**/0.114
			
Rare Hom+Het vs. Common Hom	1.096(0.939–1.279)	1.012(0.912–1.123)	1.028(0.941–1.123)
*P/Ph*	0.245/0.545	0.824/0.994	0.537/0.805
			
Rare Hom vs. Het+Common Hom	1.085(0.843–1.395)	1.154(0.988–1.347)	**1.202 (1.051–1.375)**
*P/Ph*	0.528/0.060	0.070/0.114	**0.007**/0.093

Codominant (Het vs. Common Hom); Codominant (Rare Hom vs. Common Hom); Dominant (Rare Hom + Het vs. Common Hom); and **Recessive** (Rare Hom vs. Het + Common Hom). Het: heterozygous (CT); Common Hom: common homozygous (CC); Rare Hom: rare homozygous (TT); Ph: P value of the heterogeneity Q test.

**Figure 3:**
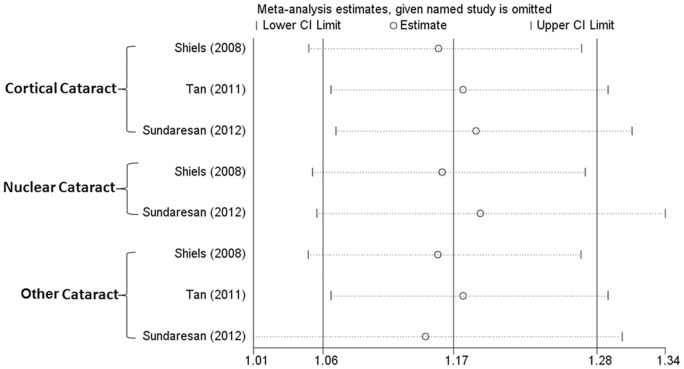
Results of the influential analysis, showing no potential influence on the overall OR in any of the cataract categories by any of the three included individual studies.

An influential analysis was also performed to determine the potential influence of each study on the overall OR. As shown in Figure 3, none of the individual studies affected the overall OR in any cataract category; omission of any single study made no substantial difference.

**Figure 4:**
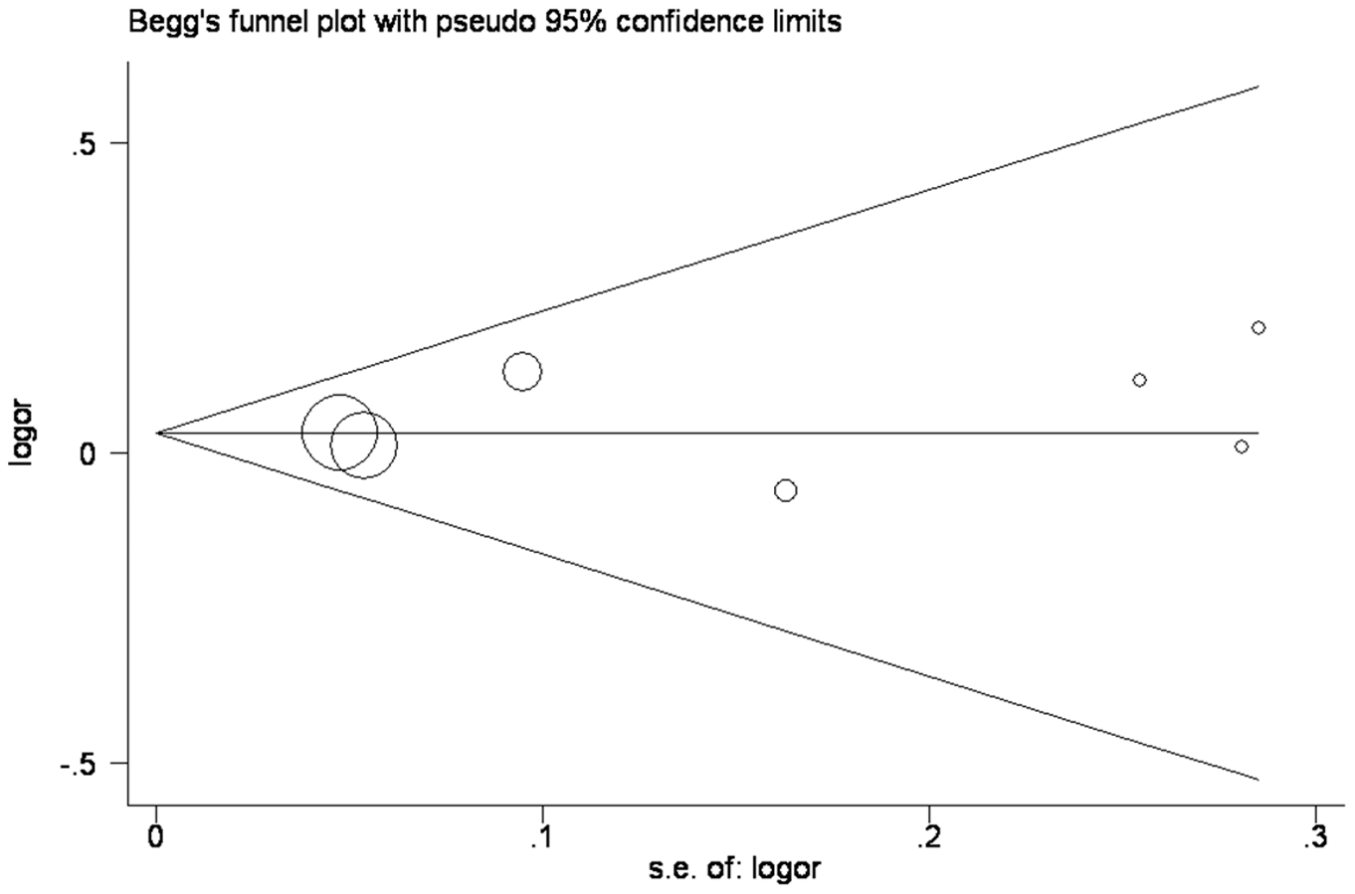
A funnel plot, showing no publication bias as assessed by Begg's funnel plot and Egger's test in this meta-analysis.

In addition, Begg's funnel plot and Egger's test were performed to evaluate the publication bias. The shape of the funnel plot did not reveal any evidence of obvious asymmetry (Fig. 4), and the Egger's test suggested an absence of publication bias for any cataracts in the recessive model (p = 0.822) and in any other genetic models.

## Discussion

Genetics has been demonstrated to play an important role in the development of both cortical and nuclear cataracts in numerous clinical studies [2], [18]. Examples include the Framingham Eye Study where strong associations were found between siblings for nuclear and posterior subcapsular cataracts [19], the Beaver Dam Eye Study where a single major gene was demonstrated to be responsible for 58% of the variable risk for the development of cortical cataract [20], and the Twin Eye Study where genetics was estimated to account for a significantly higher proportion of the risk for the development of nuclear cataract than environmental factors (48% vs 14%) [5].

As the interest in the role of genetic component in the development of cataract is growing exponentially over the past two decades, variations have been identified as putative causes of cataracts in more than 35 loci within the human genome [12]. One of these loci is in the human chromosome 1p36 region where the *EPHA2* gene is harbored [8], [9]. Early analyses linked the *EPHA2* gene with autosomal dominant cataract [21], [22] and several recent studies have attempted to determine the association between *EPHA2* and age-related cataract [14]–[16], [23]. A wide spectrum of SNPs on this gene have been assessed in these studies involving populations of different racial/ethnical backgrounds in different continents, but inconsistent results have been obtained. In this meta-analysis, we aimed to identify the “true effect size”, i.e., the *EPHA2* SNP(s) among those evaluated in previous studies that is truly associated with the risk for age-related cataract. To this end, we pooled the data from three independent clinical studies [14]–[16] that met all the inclusion criteria for this meta-analysis study and analyzed the association of rs3754334, the only polymorphism that was assessed in all the three included studies, with the risk of age-related cataract.

Our analyses showed that the polymorphism rs3754334 was associated with the risk for any age-related cataracts but neither cortical nor nuclear cataract. This result was not totally consistent with those presented individually in the three individual studies included in this meta-analysis. In the US study, minor allele frequencies for rs3754334 in cataract cases were only slightly higher than those in controls (34% vs 28%) with no significant allelic p values obtained [14]. Similarly, no association between rs3754334 and the risk of age-related cataract was demonstrated in the Indian [15] and Chinese [16] studies. Instead, highest levels of association were demonstrated between rs7543472 and cortical cataract and any age related cataracts and between rs11260867 and cortical cataract in the US study [14]; moderate levels of association were demonstrated between minor allele homozygous genotypes of rs7543472 and rs11260867 and cortical cataract and posterior subcapsular cataract but not nuclear cataract or any cataracts in the Indian study [15]; and moderate levels of association were demonstrated between rs477558 and rs7548209 and age-related cataract in the Chinese study [16]. It is well-accepted that meta-analysis increases the sample size of individual studies and thus the power of analyzing the effect of interest. We therefore believe that the polymorphism rs3754334 of the *EPHA2* gene is truly associated with the risk for the development of age-related cataract across populations of different ethnic/racial backgrounds. Nevertheless, this does not mean that the association of other polymorphisms on the *EPHA2* gene assessed in the individual studies with the age-related cataract risk is not true. It might be very likely that those associations demonstrated in the individual studies would become stronger if they were included in the meta-analysis. Unfortunately, however, no polymorphisms other than rs3754334 were assessed in all the three included studies, making the analysis of other polymorphisms impossible in this study.

The polymorphism rs3754334 is a synonymous polymorphism in *EPHA2*, which leads to 2874C->T in the *EPHA2* mRNA. However, this C->T switch does not change the protein sequence and thus rs3754334 is not a functional polymorphism. It might be possible that the rs3754334 mutation results in changes in the configuration of the *EPHA2* protein, thereby increasing the risk of age-related cataract development. Nevertheless, this has yet to be further elucidated.

Some limitations of this meta-analysis should be acknowledged. First, although the available data on rs3754334 comprised 4776 cases and 3116 controls, no association was found between this SNP and cortical cataracts. Therefore, additional study is warranted to determine the true association rs3754334 with the risk for cortical cataract. Second, the primary outcome measure was calculated based on individual unadjusted ORs. This might affect the evaluation precision of the study. Finally, the lack of individual data in each study prevented more detailed analyses on combined effects of SNP–SNP or gene–environment factors.

In summary, this meta-analysis of three individual studies on different ethnic/racial populations in different continents has shown that rs3754334 might be a polymorphism that is commonly occurring in the *EPHA2* gene of populations of different racial backgrounds and associated with the age-related cataract risk, in addition to the polymorphisms individually demonstrated in the three included studies. Further investigations are warranted to validate the role for rs3754334 in the development of age-related cataract and the underlying mechanism(s).

## Supporting Information

Checklist S1 **PRISMA 2009 checklist.**
(DOC)
